# Refractory hypokalemia and metabolic acidosis induced by undifferentiated connective tissue disease secondary to immune checkpoint inhibitors: a case report and literature review

**DOI:** 10.3389/fonc.2024.1442605

**Published:** 2024-11-26

**Authors:** Yinfang Gu, Lilan Yi, Xiaofang Zou, Longhua Guo, Guowu Wu, Jingjing Zhao

**Affiliations:** ^1^ Department of Oncology, Cancer Center, Meizhou People’s Hospital (Huangtang Hospital), Meizhou Academy of Medical Sciences, Meizhou, Guangdong, China; ^2^ Guangdong Provincial Engineering and Technological Research Center for Clinical Molecular Diagnosis and Antibody Drugs, Meizhou, Guangdong, China; ^3^ State Key Laboratory of Oncology in South China, Guangzhou, China; ^4^ Department of Biotherapy, Sun Yat-sen University Cancer Center, Guangzhou, China

**Keywords:** refractory hypokalemia, electrolyte imbalance, renal tubular acidosis, undifferentiated connective tissue diseases, immune checkpoint inhibitors, camrelizumab, malignant melanoma

## Abstract

In the past, immune checkpoint inhibitors (ICIs) like camrelizumab have been associated with rheumatic immune-related adverse events (irAEs).To prevent serious adverse consequences, early diagnosis of rheumatic irAEs is crucial. A 40-year-old patient with malignant melanoma experienced severe hypokalemia and fatigue after 6 months of camrelizumab therapy, which was unresponsive to potassium chloride supplementation. Subsequently, the patient was diagnosed with refractory hypokalemia secondary to type I renal tubular acidosis (RTA). After treatment with potassium citrate and hydroxychloroquine, blood potassium, chloride, carbon dioxide binding capacity, and arterial blood gases returned to normal and the fatigue symptoms disappeared. However, severe hypokalemia and fatigue returned following resumption of camrelizumab therapy, and only resolved upon discontinuation and intensified symptomatic treatment. No recurrence of the condition was observed after camrelizumab was discontinued. Refractory hypokalemia and RTA were attributed to undifferentiated connective tissue disease (UCTD), a rheumatic condition considered as an adverse event of camrelizumab. This case underscores the necessity of monitoring serum potassium levels during ICI therapy and the consideration of RTA and autoimmune diseases in cases of hypokalemia to prevent serious adverse consequences.

## Introduction

Immune checkpoint inhibitors (ICIs) have revolutionized the treatment of refractory tumors by blocking key inhibitory regulatory molecules on T cells, thereby triggering T cell-mediated anti-tumor responses that can lead to lasting therapeutic effects (1). The ICIs typically include programmed death-1 (PD-1) receptor inhibitors (such as nivolumab, pembrolizumab and camrelizumab), programmed cell death ligand 1 (PD-L1) inhibitors (such as atezolizumab, avelumab, and durvalumab), and cytotoxic T-lymphocyte-associated antigen 4 (CTLA-4) inhibitors (such as ipilimumab) (2). However, the inhibition of immune checkpoint can disrupt the homeostasis of the human immune system, leading to immune cells attacking the body, resulting in autoimmune side effects known as immune-related adverse events (irAEs). These can affect various systemic tissues, including the skin, gastrointestinal tract, lungs, endocrine system, kidneys, and lead to rheumatic immune-related adverse events (3, 4).

Although specific rheumatic manifestations are rarely reported as irAEs in randomized clinical trials, they appear to be significantly more prevalent in actual clinical practice (5). Compared with other immune-related adverse events, rheumatic immune-related adverse events are more challenging to estimate due to the lack of specific definitions for musculoskeletal manifestations in oncological clinical trials (5). The reported incidence of rheumatic irAEs ranges from 0.4% to 16%, with rheumatic irAEs often underreported in clinical trials (6, 7). Currently, PD-1 inhibitors are being considered as one of the most important first-line treatment options for malignant melanoma in current clinical practice (8). There has been some evidence to support the idea that PD-1 inhibitors can cause rheumatic diseases, however clinical researchers haven’t given sufficient attention to early detection and diagnosis of rheumatic immune-related adverse events (9). This article comprehensively reports a case of rheumatic irAEs with refractory hypokalemia and metabolic acidosis as the initial manifestation after treatment with a PD-1 inhibitor, and early diagnosis and treatment have prevented the occurrence of serious adverse outcomes, providing certain guiding value for the future clinical application of PD-1 inhibitors.

## Case report

A 40-year-old patient presented to the Endocrinology Department of our center on July 20, 2023, with a complaint of “fatigue for over a month.” Eight months ago, the patient was diagnosed with acral malignant melanoma (stage pT3bN0M0, stage IIB) after undergoing wide local excision of a malignant melanoma on the right plantar foot. The BRAF V600 mutation was not detected. Currently, there are no established postoperative adjuvant treatment standards for stage IIB acral melanoma without BRAF V600 mutation. Most treatments follow the standards for cutaneous melanoma, using anti-PD-1 antibodies such as pembrolizumab for one year (10). However, due to the high cost of pembrolizumab (RMB 35,000 per cycle for 17 cycles), the patient was unable to afford it and declined its use. Moreover, previous studies suggested that the response rate of acral melanoma to ICI monotherapy (8.6%-21.2%) was significantly lower than that of cutaneous melanoma (11, 12). A phase II study in China reported that camrelizumab combined with apatinib (RMB 5,000 per cycle) for first-line treatment of acral melanoma showed an objective response rate (ORR) of 24.1%, which appears to be more favorable than pembrolizumab monotherapy (ORR 15.8%) (13, 14). After being fully informed, the patient ultimately opted for the more affordable combination therapy of camrelizumab and apatinib. Seven months before, the patient began receiving intravenous infusions of camrelizumab at a dose of 200mg every 3 weeks, concurrently with oral apatinib at a dose of 250mg once daily. During the treatment, the patient experienced recurrent drug-induced liver injury, leading to an irregular use of apatinib, which was discontinued 72 days ago. The last dose of camrelizumab the patient received was 21 days ago. More than 40 days ago, the patient developed generalized fatigue without an apparent cause, graded as level 2 according to the Common Terminology Criteria for Adverse Events (CTCAE) version 5.0, without nausea, vomiting, abdominal distension, diarrhea, chills, fever, dizziness, headache, palpitations, or other discomforts (2, 15). From 30 days ago until the day before admission, the patient’s serum potassium levels fluctuated between 2.58-2.85mmol/L, graded as level 3 according to CTCAE version 5.0. The patient’s fatigue persisted despite intravenous and oral potassium chloride therapy, causing the patient to discontinue camrelizumab treatment. The patient was admitted for further evaluation and management of refractory hypokalemia. The patient had no history of autoimmune diseases, hypertension, diabetes, heart disease, or food/drug allergies. Recently, there is no history of use of other medications such as proton pump inhibitors (PPIs) or Non-Steroidal Anti-inflammatory Drugs (NSAIDs).

Upon admission, the patient’s vital signs were stable, with a clear consciousness, normal breathing, elastic skin, no rash, smooth oral mucosa, normal limb joint movement without pain, and normal muscle strength. From 30 days ago until the day before admission, laboratory findings revealed fluctuations in serum potassium (2.58-2.85mmol/L), chloride (111.0-115.1mmol/L), carbon dioxide binding capacity (13.2-16.2mmol/L), the serum anion gap was normal, creatine kinase (CK) levels were normal, and urine pH 7-7.5. Thyroid function tests 30 days ago showed normal levels of thyroid-stimulating hormone (TSH), free triiodothyronine (FT3), and free thyroxine (FT4), with normal cortisol levels. On the day before admission, biochemical analysis revealed urea and creatinine levels of 4.57 mmol/L and 70.5 µmol/L, respectively, both within the normal range. Thyroid function tests showed a slightly elevated TSH level of 5.018 uIU/mL (upper normal limit of 4.94 uIU/mL), while FT3 and FT4 levels remained normal. A routine electrocardiogram revealed sinus tachycardia and T-wave changes, while echocardiography showed no significant abnormalities.

Diagnoses upon admission were hypokalemia (grade 3) and acral malignant melanoma. From the day of admission, the patient received intravenous and oral potassium chloride supplementation. the patient received intravenous and oral potassium chloride supplementation. On the day of admission and the following day, further investigations showed a normal cortisol rhythm and an adrenocorticotropic hormone (ACTH) rhythm that was mostly normal, with 8 AM and 4 PM levels within the normal range, and a slightly low midnight level of 6.52 pg/mL (normal lower limit: 7.2 pg/mL). The Reproductive Hormone Panel indicated that Follicle-Stimulating Hormone (FSH), Luteinizing Hormone (LH), Prolactin (PRL), Estradiol (E2), Progesterone (P), and Testosterone (T) were all within normal ranges. At the time of admission, the patient only presented with grade 2 fatigue and did not exhibit other symptoms associated with hypophysitis, such as headache or polyuria. Blood pressure was normal, and pre-admission tests suggested hypokalemia unresponsive to potassium chloride treatment, with no evidence of hyponatremia. Since the Reproductive Hormone Panel, thyroid function, cortisol rhythm, and ACTH rhythm were all within normal ranges, with no clear reduction in pituitary hormone levels, hypophysitis was excluded as a diagnosis (16).

Two days after admission, arterial blood gas analysis showed a pH of 7.34, partial pressure of carbon dioxide of 23mmHg, partial pressure of oxygen of 54mm Hg, oxygen saturation of 85.9%, actual bicarbonate of 12.2 mmol/L, standard bicarbonate of 15.4mmol/L, and total carbon dioxide of 12.9mmol/L. A 24-hour urine biochemistry revealed sodium at 78 mmol/L, elevated potassium at 33.96 mmol/L, chloride at 91.1 mmol/L, and calcium at 1.85 mmol/L. Based on recent findings, the patient had grade 2 fatigue and was diagnosed with refractory hypokalemia, increased urinary potassium excretion, hyperchloremia with metabolic acidosis, and alkaline urine. Creatinine levels were normal, ruling out extrarenal causes such as diarrhea and pancreatic leakage. The presence of alkaline urine excluded type II and type IV renal tubular acidosis (RTA), suggestive of type I RTA (16). In order to supplement potassium, the patient was given potassium citrate granules (1 packet tid).

Reports of ICI-induced RTA are rare. In patients without a history of kidney disease, potential causes of RTA include medications, autoimmune conditions such as Sjögren’s syndrome or rheumatoid arthritis (RA), post-kidney transplant complications, nephrocalcinosis, medullary sponge kidney disease, chronic urinary tract obstruction, liver cirrhosis, and sickle cell anemia (17). First, the patient did not present with an acute onset of symptoms, creatinine levels were normal, and there was no presence of urinary white blood cells, hematuria, or proteinuria. There was no history of primary type I RTA, such as osteopetrosis, Wilson’s disease, or carbonic anhydrase deficiency, nor was there any recent infection. Additionally, there was no concurrent medication use that could cause RTA (apatinib has a half-life of 8.5-9.0 hours, and the patient had already stopped using apatinib 42 days prior to the onset of grade 3 hypokalemia. Furthermore, apatinib is not associated with adverse events like metabolic acidosis or interstitial nephritis, making apatinib-related RTA unlikely. The patient also had no recent use of NSAIDs, PPIs, or other medications). There was no evidence of hypercalciuria, hyperparathyroidism, or calcium metabolism disorders, nor a history of obstructive nephropathy or any underlying diseases that could lead to interstitial nephritis. Therefore, other potential causes of RTA, apart from autoimmune conditions, were largely excluded.

On the third day after admission, quantitative determination of anti-double-stranded DNA antibodies and anti-nuclear antibodies (ANA) showed positive results, with a titer of 1:100. The spectrum of anti-nuclear antibodies showed strong positivity for anti-SSA antibodies and anti-Ro-52 antibodies, while the rest were negative. Tests for vasculitis antibodies and vasculitis target antigens, as well as complement immunoglobulins, showed no significant abnormalities. Thus, ICI-related autoimmune disease secondary to RTA was considered. The strong positivity for anti-SSA antibodies and anti-Ro-52 antibodies suggested the need to differentiate conditions such as Sjögren’s syndrome, systemic lupus erythematosus (SLE), scleroderma, and myositis, which are all rheumatic autoimmune diseases. Since the patient had no pain, skin, or joint abnormalities, and creatine kinase, anti-Scl-70 antibodies, and anti-centromere antibodies were within normal levels, immune arthritis, myositis, myalgia, and scleroderma were excluded. Additionally, the absence of symptoms such as rash, erythema, photosensitivity, oral ulcers, or arthritis, along with negative urine protein, normal blood cell counts, and negative anti-dsDNA antibodies, anti-Sm antibodies, and antiphospholipid antibodies, ruled out lupus. Therefore, the focus shifted to determining whether a diagnosis of Sjögren’s syndrome could be confirmed (18).

**Figure 1:**
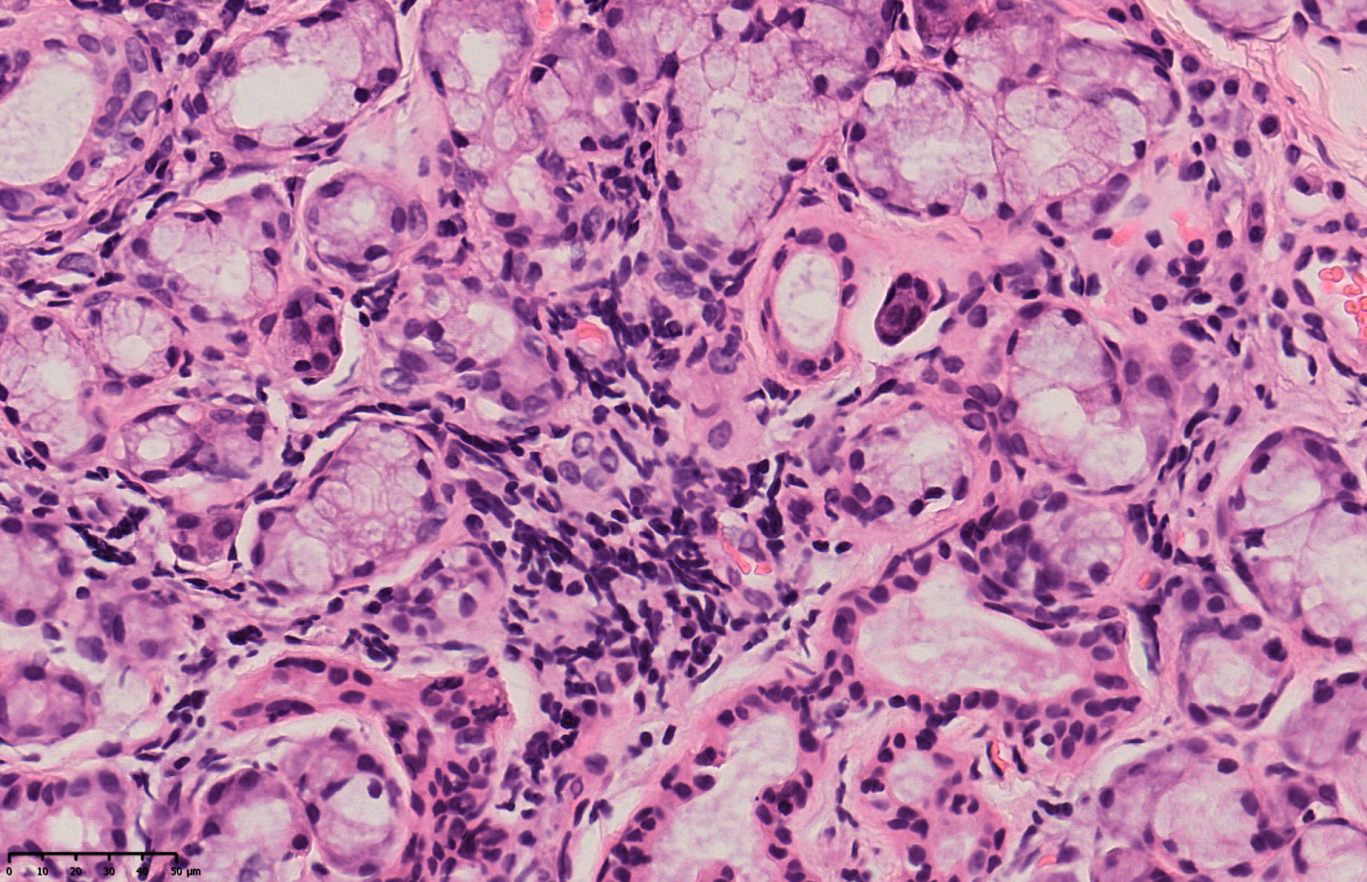
Lip gland biopsy pathology report: (lip gland) examined salivary gland tissue showed scattered lymphocyte infiltration.

On the fourth day after admission, dry eye screening revealed Tear film breakup time (TBUT): Oculus Sinister (OD) 21.6s (first time), average 23.73s, grade 0; Oculus Sinister (OS) 7.07s (first time), average 13.80s, grade 1. Salivary gland scintigraphy showed normal uptake function of bilateral parotid glands but impaired secretion function. On the sixth day after admission, the patient’s serum potassium was 2.88mmol/L. The dosage of potassium citrate granules was adjusted to 2 packets tid, resulting in a gradual increase in serum potassium and the resolution of fatigue symptoms. The patient was discharged on the seventh day after admission. On the eighth day after admission, the lip gland biopsy pathology report showed mild lobular atrophy and scattered lymphocyte infiltration in the examined salivary gland tissue (**Figure 1**). Although the patient showed strong positivity for anti-SSA antibodies and anti-Ro-52 antibodies, there were no typical symptoms of dry mouth or dry eyes. The lip gland biopsy pathology, salivary gland scintigraphy, and tear film break-up time showed mild abnormalities, but did not meet the diagnostic criteria for Sjögren’s syndrome. After consulting with rheumatology experts, it was concluded that the patient had no prior history of rheumatic autoimmune diseases and had a history of ICI use. The patient developed RTA secondary to grade 3 hypokalemia, and acute interstitial nephritis was largely excluded. Immunological testing showed ANA positivity and strong positivity for anti-SSA and anti-Ro-52 antibodies, and mild abnormalities were detected in the lip gland biopsy, salivary gland scintigraphy, and tear film break-up time, indicating mild damage. Notably, the lip gland biopsy revealed mild lobular atrophy and scattered lymphocyte infiltration in the examined salivary gland tissue, suggesting mild immune-related damage. Other well-defined connective tissue diseases, such as SLE, rheumatoid arthritis, Sjögren’s syndrome, polymyositis, and dermatomyositis, were ruled out. Given that ICI-induced rheumatic autoimmune diseases often present with a disease course and clinical manifestations different from those of previously diagnosed autoimmune conditions (2), a diagnosis of undifferentiated connective tissue disease (UCTD) was made, with consideration of its association with ICI therapy.

**Figure 2:**
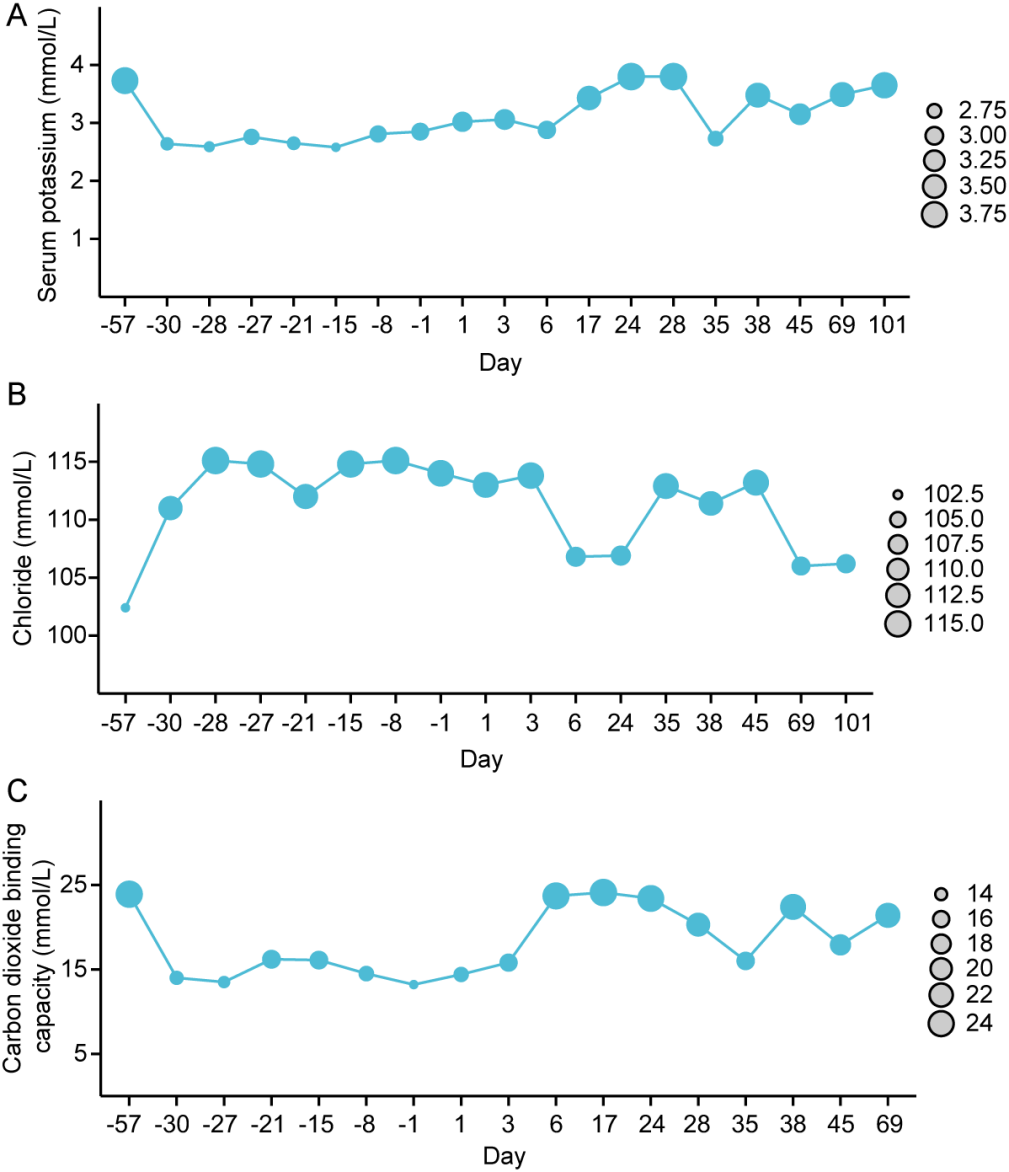
The dynamic changes in blood potassium **(A)**, blood chloride **(B)**, and carbon dioxide binding capacity level **(C)**, (Day 0 refers to the day of admission).

Continuous monitoring revealed that, despite treatment with potassium citrate granules, the patient’s blood potassium levels remained at grade 1 hypokalemia. After consulting with in-hospital rheumatology experts and considering the findings from Roberts et al. (19), it was concluded that the patient’s fatigue was only grade 2, with no signs of significant organ dysfunction typically associated with grade 3 hypokalemia, aside from mild ECG abnormalities. Given the relatively mild symptoms, the patient’s symptoms resolved after treatment with potassium citrate granules, and only grade 1 hypokalemia persisted. It was recommended that hydroxychloroquine might be an effective treatment with fewer side effects compared to corticosteroids. Immunomodulatory therapy with hydroxychloroquine 200mg bid was initiated on the 17th day after admission. One week after treatment, serum potassium normalized. On the 28th day after admission, electrolytes, liver and kidney function, arterial blood gas analysis, electrocardiogram, and echocardiography were all within normal ranges. The administration of camrelizumab was resumed on the 29th day after admission. However, following treatment, the patient experienced fatigue (grade 2) symptoms again. On the 35th day after admission, the patient’s serum potassium was 2.73mmol/L (grade 3) and chloride was 112.9 mmol/L (tested at a local clinic near the patient’s home, without returning to the hospital). Considering signs of disease recurrence, she received intensified treatment under telephone guidance. The serum potassium gradually increased after oral sodium bicarbonate solution, potassium citrate supplementation and continuous oral hydroxychloroquine tablets. The dynamic trends of blood potassium, blood chloride, and bicarbonate levels are illustrated in **Figures 2A–C**. Considering the recurrence of grade 3 hypokalemia despite treatment, camrelizumab was permanently discontinued. In addition, the patient needs to continue maintenance treatment with potassium citrate and hydroxychloroquine with regular follow-up. The patient has not experienced any further episodes of metabolic acidosis or grade 2 or higher hypokalemia, and no adverse reactions to the medications have been reported. Compliance has been good, and the use of corticosteroids and their potential side effects have been avoided. After discontinuation of adjuvant therapy for the primary disease, malignant melanoma, the patient has undergone regular follow-up, and thus far, over a year post-surgery, there have been no signs of tumor recurrence or metastasis.

The refractory hypokalemia induced by RTA in the patient was considered to be attributable to ICI treatment, based on the following factors: the adverse event (AE) occurred after the use of camrelizumab, indicating a temporal association; there have been previous reports of rheumatic irAEs with ICIs, which are recognized as known drug-related side effects. The patient experienced grade 3 hypokalemia 42 days after discontinuing apatinib, a drug with a half-life of 8.5-9.0 hours, and apatinib is not associated with reported AEs such as metabolic acidosis or interstitial nephritis. Therefore, apatinib-related causes were ruled out. Additionally, the patient had no other medications, such as NSAIDs or PPIs, that could explain the condition. After discontinuation of ICI therapy, the patient’s condition gradually stabilized without further exacerbation, meeting the criterion of dechallenge in AE assessment. Upon reintroduction of camrelizumab, grade 3 hypokalemia recurred, but resolved after intensified potassium and bicarbonate supplementation, fulfilling the criterion of rechallenge in AE evaluation. Based on what have been discussed above, we concluded that the AE was associated with ICI therapy (20).

## Discussion

The emergence of ICIs has revolutionized the landscape of cancer therapy and become an effective treatment modality for malignant melanoma (21). An increasing number of reports have been published about immune checkpoint inhibitor side effects, some of which may be severe or even life-threatening, the European League Against Rheumatism (EULAR) states that Due to the absence of specific definitions for musculoskeletal manifestations, diagnosing rheumatic irAEs is more challenging in clinical practice, which can result in underdiagnosis and potentially adverse outcomes and recommends that the diagnosis and management of rheumatic irAEs should be a shared decision between patients, oncologists, and rheumatologists (2). Therefore, this case report emphasizes the importance of diagnosis and early detection of a rheumatic irAE to prevent serious adverse outcomes.

The patient in this case had no history of rheumatic or immune-related diseases. Following treatment with an PD-1 inhibitor for malignant melanoma, the patient developed refractory hypokalemia that was unresponsive to potassium chloride infusion and oral supplementation, graded as CTCAE 5.0 Grade 3, accompanied by CTCAE 5.0 Grade 2 fatigue and no other discomfort, tests of the Reproductive Hormone Panel, thyroid function, cortisol rhythm, and ACTH rhythm ruled out hypophysitis. Laboratory tests revealed hypokalemia, hyperchloremia with metabolic acidosis, alkaline urine, normal creatinine, and urea nitrogen levels, leading to a diagnosis of type I RTA. Based on the patient’s medical history, concomitant medications, symptoms, signs, and existing test results, drug-related causes, infections, and other primary diseases were ruled out. The patient had no prior history of rheumatic autoimmune diseases but had a history of ICI use, which led to the development of RTA and grade 3 hypokalemia, while acute interstitial nephritis was largely excluded. In adults, type I RTA is typically caused by autoimmune diseases affecting the kidneys (22). Further laboratory examinations showed strongly positive anti-SS-A and anti-Ro-52 antibodies, with mild abnormalities in lip gland biopsy, salivary gland scintigraphy, and tear film break-up time. The combination of the patient’s clinical presentation and examination results ruled out other well-defined connective tissue diseases such as SLE, rheumatoid arthritis, Sjögren’s syndrome, polymyositis, and dermatomyositis. Since the course and clinical manifestations of ICI-induced rheumatic autoimmune diseases often differ significantly from those of previously diagnosed autoimmune conditions, a final diagnosis of UCTD related to ICI use was made. The etiology of autoimmune distal RTA is attributed to impairments in the proton pump of the distal tubule, which can be caused by immunological damage or the presence of autoantibodies (23). Given the role of PD-1 signaling in regulating T-cell-mediated renal inflammation (24), it is plausible that anti-PD-1 therapies could induce immunologic damage similar to that seen in autoimmune conditions, potentially leading to RTA. This suggests the presence of early secondary UCTD with RTA-induced hypokalemia.

Potassium citrate supplementation and hydroxychloroquine immunomodulatory therapy normalized potassium, chloride, bicarbonate levels, arterial blood gases, etc. However, resuming therapy with the PD-1 inhibitor resulted in a recurrence of CTCAE 5.0 Grade 3 hypokalemia with Grade 2 fatigue. The refractory hypokalemia appeared again soon with the treatment of PD-1 inhibitor, consistent with the instructions and guidelines documented risk of hypokalemia and secondary autoimmune diseases associated with ICIs. Previously reported cases of severe hypokalemia, RTA, and limb paralysis induced by nivolumab were considered as irAEs (25). In this patient, the refractory hypokalemia is thus considered as irAEs induced by the camrelizumab.

Camrelizumab is a monoclonal antibody targeting programmed death protein 1 (PD-1) receptor widely utilized in anti-tumor therapy (26). Hypokalemia is a common adverse reaction to PD-1 inhibitors, mostly mild to moderate and correctable with oral or intravenous potassium supplementation. However, severe and refractory hypokalemia caused by camrelizumab has not been reported. Three cases of acute interstitial nephritis secondary to RTA associated with ICIs have been reported, all accompanied by elevated creatinine levels and showing improvement after steroid treatment (27). El Bitar S et al. also reported one case of RTA secondary to nivolumab, with elevated creatinine levels that responded rapidly to steroids (28). The condition of Sjögren’s syndrome linked to immune checkpoint inhibitors, which accounts for 0.3 to 2.5 percent of cases, is often overlooked in diagnosis and may result in serious systemic complications (29). Acute interstitial nephritis induced by ICIs typically presents with metabolic acidosis and hypokalemia, often accompanied by an acute rise in creatinine levels. Clinicians should consider further investigations when evaluating patients with these symptoms, as most cases respond well to steroid therapy. Anson D et al. reported a case of severe hypokalemia attributed to immune enteritis (30). A case of pathology-confirmed isolated distal tubular lesion has been reported, suggesting ICI-related isolated renal tubular acidosis without acute kidney injury, immunology-related autoantibody tests were negative (31). A case of lupus-like nephritis was linked to the administration of ICI (32). This highlights the importance of carefully ruling out other potential ICI-related or unrelated diagnoses when encountering patients with severe hypokalemia and metabolic acidosis, and of providing timely treatment to prevent adverse outcomes. In our case, the patient presented with refractory CTCAE Grade 3 hypokalemia that was unresponsive to potassium chloride therapy, later diagnosed as RTA secondary to UCTD. Treatment of rheumatic irAEs includes symptomatic supportive care and consideration of corticosteroids and immunomodulators such as hydroxychloroquine and methotrexate (2). According to Roberts J et al., hydroxychloroquine may limit glucocorticoid exposure in immune checkpoint inhibitor-induced inflammatory arthritis (19). Following treatment with hydroxychloroquine, our patient’s symptoms were quickly controlled, thus avoiding the need for prolonged steroid use and preventing the associated serious adverse effects and complications of long-term steroid use. Prompt diagnosis and treatment prevented serious adverse outcomes, unlike other reported cases that necessitated ICU admission due to the severity of the adverse events. To the best of our knowledge, this is the first reported case where refractory hypokalemia and metabolic acidosis serves as the initial manifestation of rheumatic immune-related adverse effects caused by a PD-1 inhibitor, suggesting the importance of early detection of rheumatic immune-related adverse effects and providing certain guiding significance for the clinical application of PD-1 inhibitors.

In conclusion, this case highlights that electrolyte disturbances attributable to ICIs may occur before typical renal toxicity symptoms such as elevated serum creatinine and urea nitrogen or classic autoimmune disease manifestations like joint pain, rash, and dry mouth. If hypokalemia is detected during immune checkpoint inhibitor treatment, attention should also be paid to whether the patient is complicated with hyperchloremia, low carbon dioxide binding capacity and alkaline urine. If present, consideration should be given to RTA and further evaluation for ICI-induced autoimmune diseases such as Sjögren’s syndrome and UCTD. Early diagnosis and treatment are paramount in preventing serious adverse outcomes.

## Data Availability

The original contributions presented in the study are included in the article/supplementary material. Further inquiries can be directed to the corresponding authors.
